# Arthroscopic Treatment of Combined Anterior and Posterior Cruciate Ligament Avulsion Fracture: A Case Report

**DOI:** 10.7759/cureus.69883

**Published:** 2024-09-21

**Authors:** Apostolos Gantsos, Christos Konstantinidis, Ioannis Vasiadis, Alexandros Eleftheropoulos, Dimitrios Giotis

**Affiliations:** 1 Orthopaedics and Traumatology, General Hospital of Naoussa, Naousa, GRC; 2 Orthopaedics, General Hospital of Ioannina "G. Hatzikosta", Ioannina, GRC

**Keywords:** all-arthroscopic fixation, anterior cruciate ligament, avulsion fracture, internal fixation, posterior cruciate ligament

## Abstract

Anterior cruciate ligament (ACL) avulsion fractures without any concomitant injuries are extremely rare in skeletally mature patients and sporadically reported in the literature. Such injuries are more likely to be associated with tibial plateau fractures, other knee ligament ruptures, distal femoral fractures, or knee dislocations. In this article, a case of a 55-year-old male, who suffered a combined displaced ACL-posterior cruciate ligament (PCL) avulsion fracture, without any related injuries, is reported. The fracture was treated all arthroscopically with both an internal fixation and a single-tunnel suture fixation. In this case, arthroscopic anatomic reduction and internal fixation of the displaced tibial eminence was achieved, and internal fixation was performed with two antegrade cannulated screws with additional single-tunnel trans-ACL suture fixation. Six months post-injury, the patient had no signs of post-traumatic osteoarthritis, restoration of range of motion, no pain, and no residual instability of the knee joint. The mechanism of this rare injury, the method of fixation that was used, and the rehabilitation protocol that was followed are demonstrated. Additionally, the clinical outcome in terms of certain parameters is evaluated. Finally, we highlight the importance of this method for additional strength of fixation and the role of early mobilization to establish satisfactory results.

## Introduction

Avulsion fracture of the anterior cruciate ligament (ACL) from the tibial eminence is an uncommon traumatic situation which mainly concerns paediatric and early adolescent population [1-4]. In such skeletally immature patients, ACL tears are extremely rare because elastic ligaments are more powerful than their osseous attachments and therefore knee injuries regarding ACL usually result in avulsion fractures [5,6]. However, these fractures can also occur in adults instead of ACL ruptures but with a much lower frequency, and usually, they are associated with concomitant damages to adjacent structures (menisci, cartilage) [2,4].

Regarding injury mechanism, anterior tibial eminence fractures might be caused by excessive rotational force applied to a hyperextended knee like when falling from a bicycle or motor vehicle or in contact sports and other stressful activities [4,7]. In cases of inadequate reduction or absence of bone union, they can cause not only knee joint laxity and instability but also extension and flexion limitation of range of motion [4,7].

Meyers and McKeever first classified these fractures into three types according to the displacement of avulsion: fractures with minimal or no displacement (type I), with partial displacement, where the anterior segment of the fragment is elevated but hinged (type II), and with complete displacement (type III) [8]. Zaricznyj [9] modified this categorization to include comminuted fractures by adding type IV: fractures with complete displacement and fragment comminution.

Type I fractures are commonly treated conservatively with immobilization of the knee joint in 10-20° of flexion and not in full extension to avoid over-tension of ACL and popliteal vessels for 6-12 weeks depending on the healing rate and radiographic findings [2]. However, for the other types, anatomical reduction and stabilization are required, which especially for types III and IV can hardly be achieved non-operatively [2,7,10]. Traditionally, open reduction and internal fixation was the first choice for the treatment of unstable types [2,7]. Nowadays, the familiarization of orthopaedic surgeons, with arthroscopy and its advantages regarding decreased morbidity, better intra-articular visualization, accurate reduction of an avulsion fracture, removal of loose fragments, and lower risk of arthrofibrosis, has led arthroscopic-assisted or all-arthroscopic reduction and fixation to become very popular [2,10,11].

Arthroscopic suture fixation systems which include suture loop transport­ers, retrograde guides, multiple drill tunnels, tissue penetrators for suture pas­sage, suture anchors, EndoButtons, or other various systems for meniscal or rotator cuff repair have been reported as techniques for the treatment of ACL avulsion fracture with promising results [10-13]. Such fractures of the posterior cruciate ligament (PCL) are relatively rare and can be easily missed, resulting in knee instability and arthritis development [14]. The combined avulsion fracture of both ACL and PCL in the same knee is an extremely rare traumatic condition, and only sporadic cases have been reported in the literature [15,16]. The purpose of our study was to report a combined displaced ACL and PCL avulsion fracture with comminution in a middle-aged man, which was treated successfully with an all-arthroscopic fixation technique.

## Case presentation

We report a 55-year-old male with severe pain in his right knee after falling from 1 m height. Apart from knee damage, physical examination did not reveal any concomitant injuries. The patient sustained a painful knee swelling whilst his joint was blocked in extension. No palpable crepitus was obvious during palpation. Moreover, although dorsalis pedis and posterior tibialis arteries were palpable, the popliteal artery could not be palpated because of the large swelling. Neural structures in the area were intact, and ankle and hip joints were free of motion.

**Figure 1:**
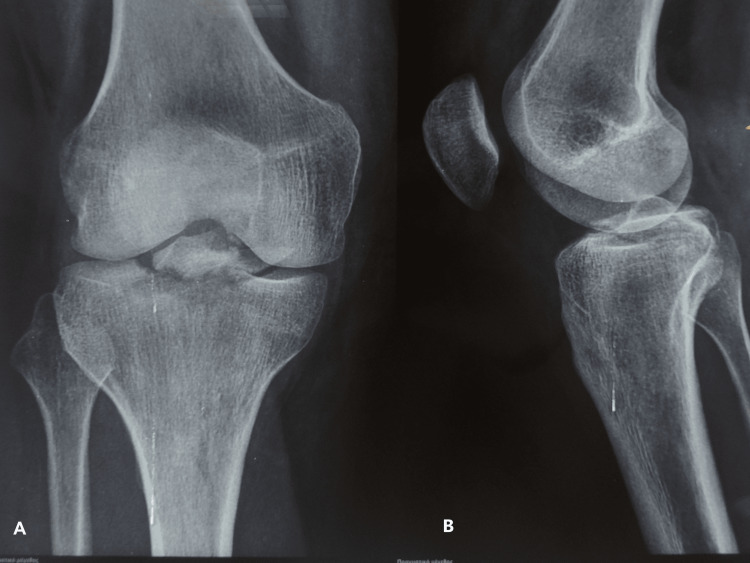
Right knee X-ray. (A) Anteroposterior view. (B) Lateral view. Patient's radiographs depicting the ACL-PCL avulsion fracture. ACL: anterior cruciate ligament; PCL: posterior cruciate ligament

Radiological control displayed a tibial eminence fracture of the right knee with a posterior extension under the PCL attachment to the tibia (Figure 1).

**Figure 2:**
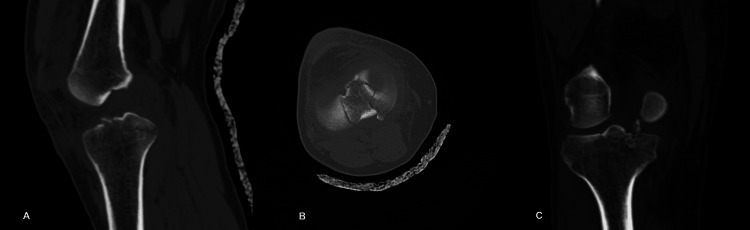
Patient's right knee CT. (A) CT sagittal view. (B) CT transverse view. (C) CT coronal view. CT: computed tomography

No other fracture was identified in the X-rays. A computed tomography (CT) scan, which was performed in order to set the diagnosis of a tibial eminence ACL avulsion fracture and to plan possible treatment options, revealed that the fracture showed a comminution in the area of the tibial eminence with a posterolateral extension but without any other intra-articular fracture (Figure 2A-2C).

**Figure 3:**
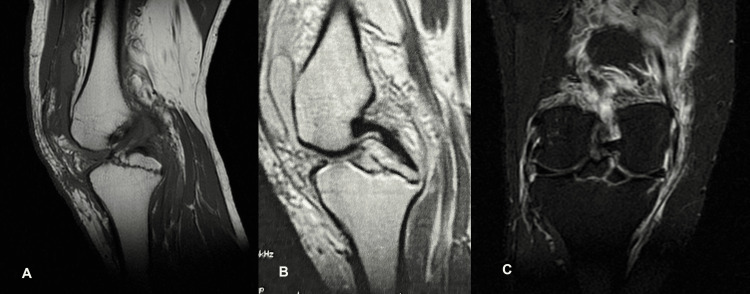
MRI of the patient's injured knee displaying the ACL-PCL avulsion fracture. (A-B) MRI sagittal view. (C) MRI coronal view. MRI: magnetic resonance imaging; ACL: anterior cruciate ligament; PCL: posterior cruciate ligament

A complementary magnetic resonance imaging (MRI) in order to check significant knee structures such as menisci, cruciate and collateral ligaments, articular cartilage, medial patellofemoral ligament (MPFL), and PCL, or presence of any bone bruise, showed that the tibial eminence was displaced upwards without any additional coronal or sagittal displacement. The fibers of ACL were intact, but they were adherent to tibial attachment although this attachment was on the avulsed fragment of the tibial spine. Likewise, PCL retained its tibial footprint to the avulsed tibial eminence (Figure 3A, 3B). In addition, the medial meniscus displayed a little horizontal rupture, the medial collateral ligament (MCL) displayed a partial one (grade 2), and there was a lateral femoral condyle bone bruise.

Surgical technique

A standard knee arthroscopy was performed under general anesthesia. The patient was placed in a supine position on the operating room table with a leg halter under the operative leg, and a tourniquet was insufflated to 300 mmHg. A standard anterolateral portal was made, and a sufficient lavage of the injured knee was performed in order to achieve an appropriate and clear view of the knee joint during the diagnostic arthroscopy and to assess intraoperatively the extent and the displacement of the fracture and the condition of all critical structures, particularly the integrity of ACL and PCL and their attachments. In order to avoid excessive bleeding and to obtain a satisfactory view of the joint, the fluid pump was set between 60 and 80 mmHg.

After thorough evacuation from hematoma and prior to the estimation of fracture extension, a diagnostic arthroscopy was performed, and a shaver (Storz Aggressive Cutter No 4.5, Germany) was used to debride the soft tissue which prohibited visualization. Concomitant injuries such as a small radial tear of the posterior horn of the medial meniscus were addressed properly before the fixation of the fracture, and the integrity of the cruciate ligament was assessed with the aid of a probe. The remaining clots were removed with the aid of a probe and blunt trocar, and the extension of the fracture was estimated.

Regarding the fracture, it was observed that there was a limited comminution between ACL and PCL attachments on the tibial spine. With the use of a probe, a vigorous trial reduction was performed by initially placing the knee gently in extension and, afterwards, under direct visualization, after elevating the medial meniscus with a blunt trocar, by pushing the fragments with the probe into their anatomical position.

**Figure 4:**
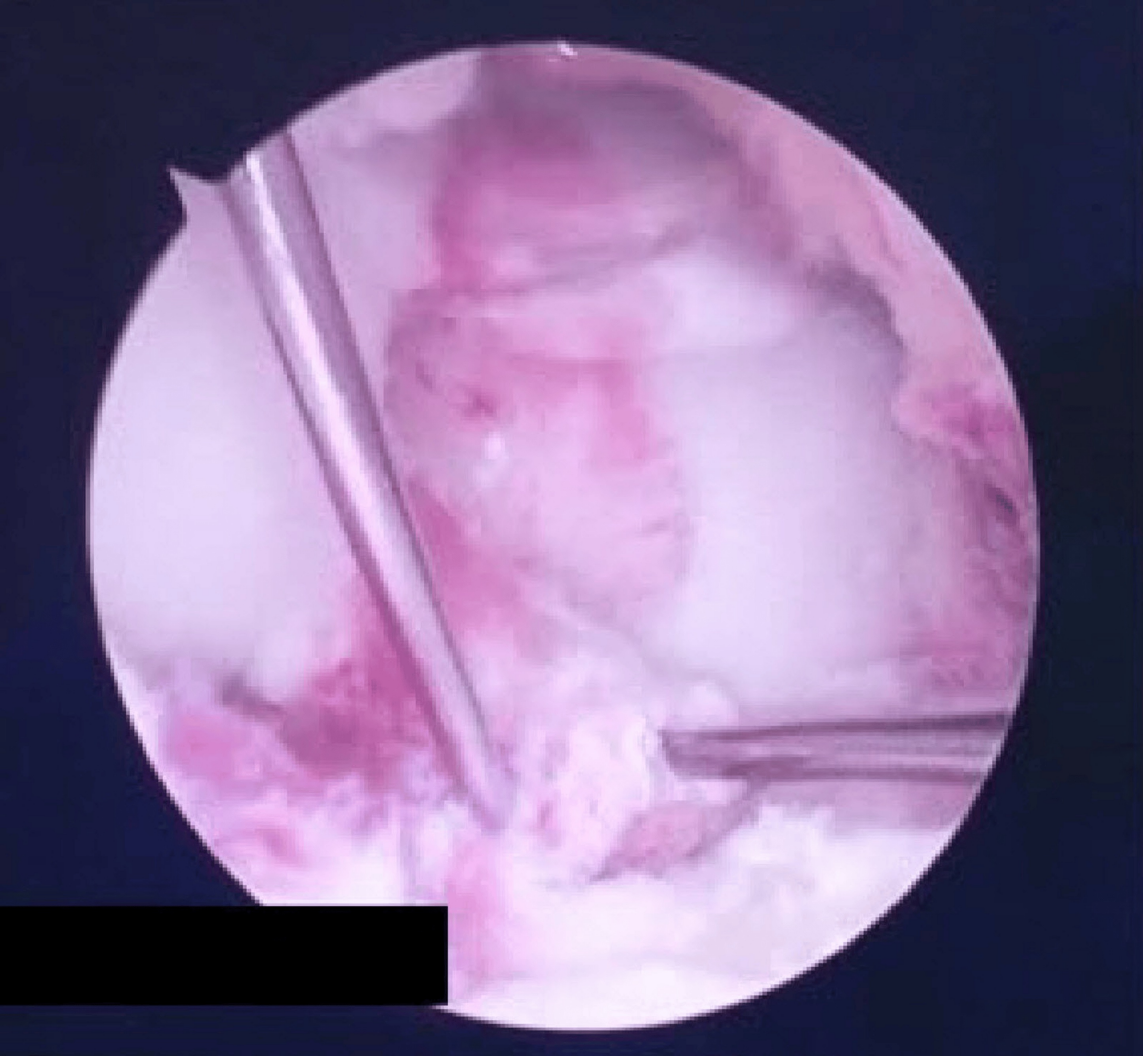
Placement of the two K/W through the avulsed fragments: arthroscopic view. K/W: Kirschner wires

After accomplishment of the trial reduction procedure under arthroscopic control, two Kirschner wires (K/W) were inserted directly into the two main fragments under direct arthroscopic visualization through separate medial and lateral high accessory portals in order to secure the result (Figure 4).

**Figure 5:**
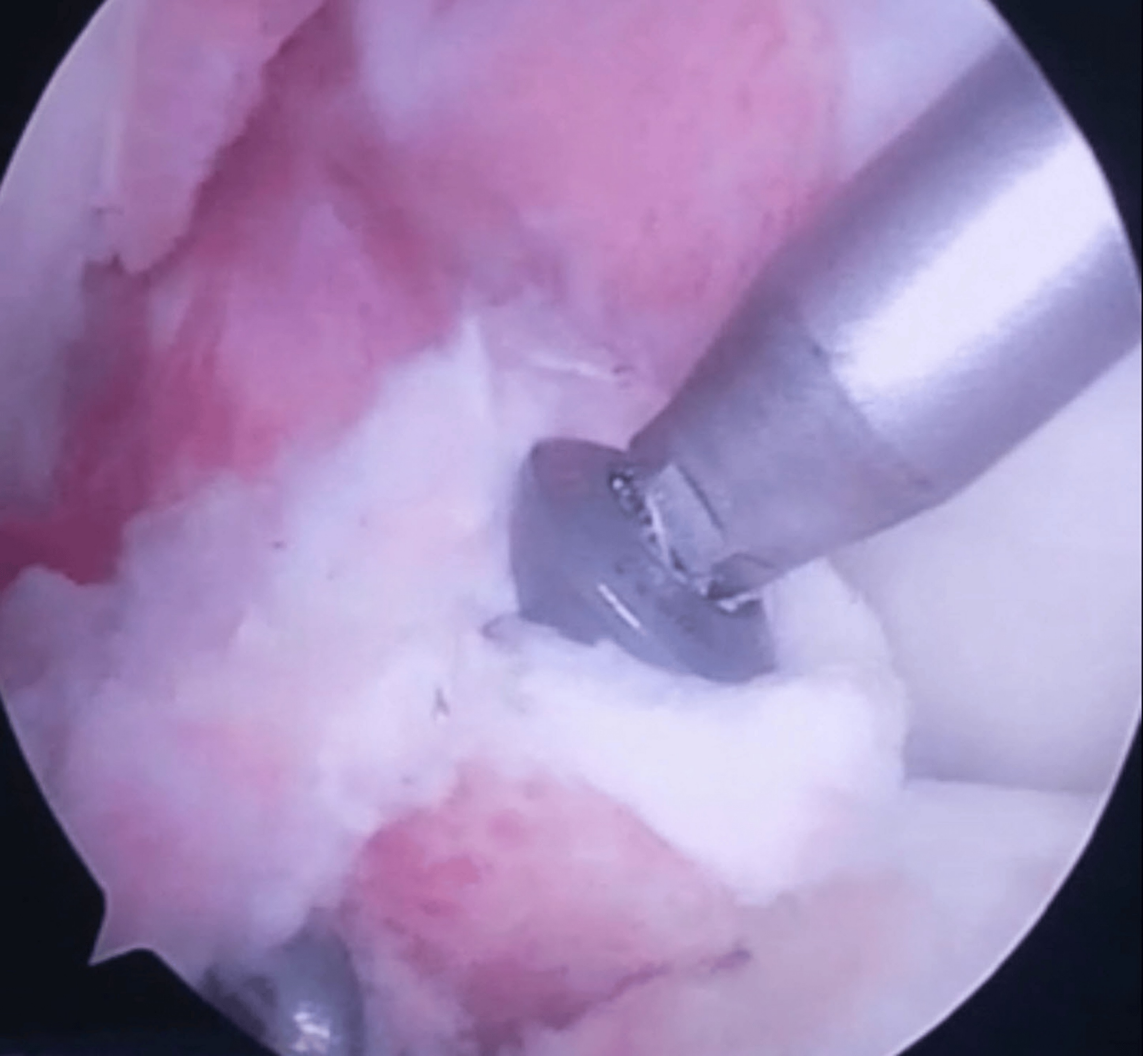
Cannulated screw insertion via K/W into the avulsed fragment. K/W: Kirschner wires

Subsequently, two cannulated screws 4 mm (Asnis 4 × 46 mm TL 15.5, 4 × 34 mm TL 11.5 mm cannulated screws, Stryker Trauma AG, Kalamazoo, MI) were inserted via K/W into the two fragments which contained the ACL and PCL attachments so as to accomplish the final internal fixation (Figure 5).

**Figure 6:**
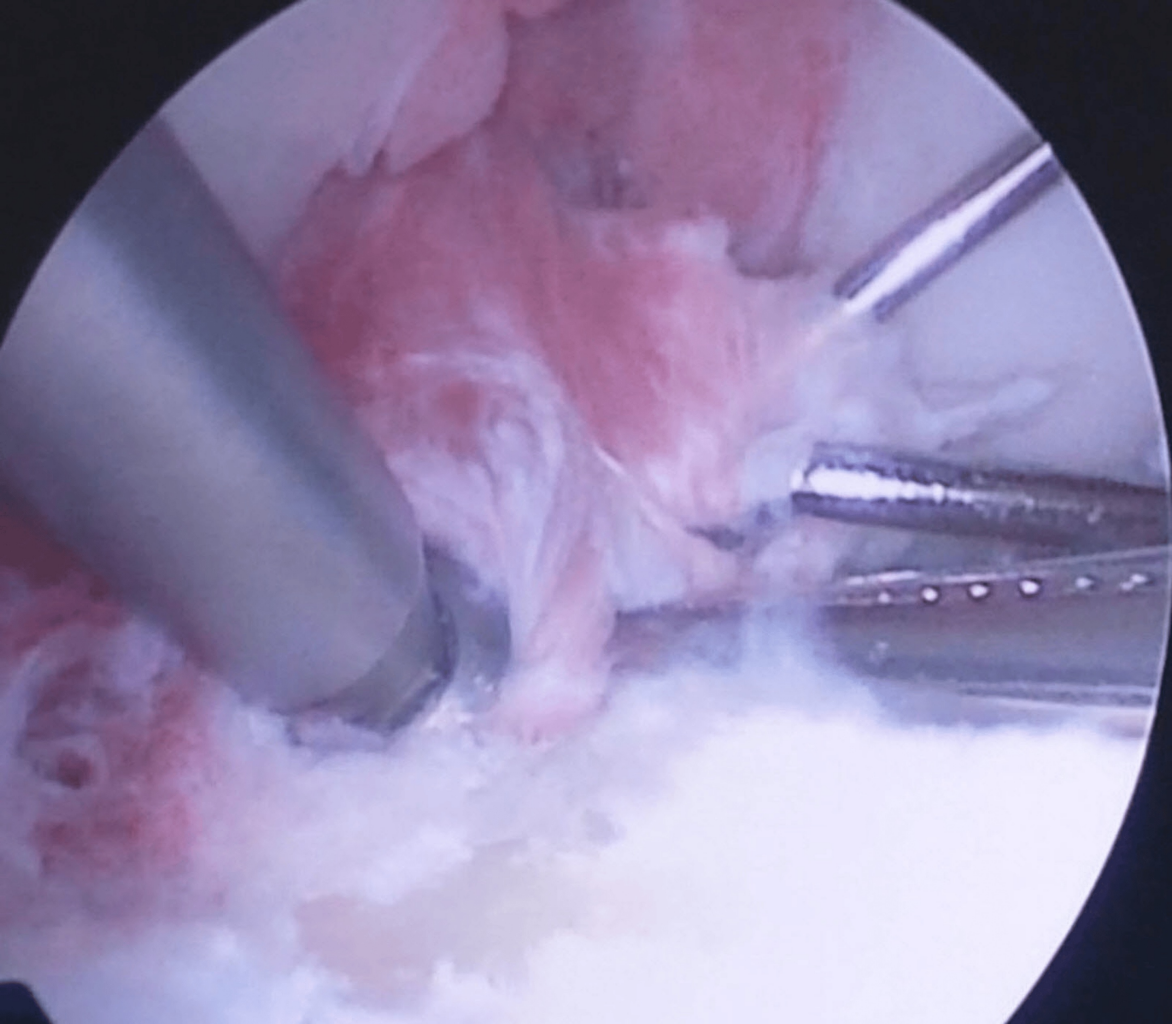
A suture passer loaded with a No 2-0 Polydioxanone suture, advanced into the joint, piercing the ACL fibers. ACL: anterior cruciate ligament

After this step, a suture passer (DePuy Mitek, Inc., Raynham, MA) loaded with a No 2-0 Polydioxanone suture (PDS Ethicon, Somerville, NJ), which was used as a guide suture, was advanced into the joint through the anteromedial portal and pierced the ACL fibers (Figure 6). The PDS suture was introduced into the joint and with a suture grasper was retrieved out of the joint through the same portal. A No 2 Orthocord suture was advanced into the joint via the guide suture and through the ACL fibers, and the limbs of this suture were secured out of the joint with a hemostat. Additionally, one more suture like the previous was introduced through the base of the ACL fibers via the same technique and secured with the same way out of the joint.

**Figure 7:**
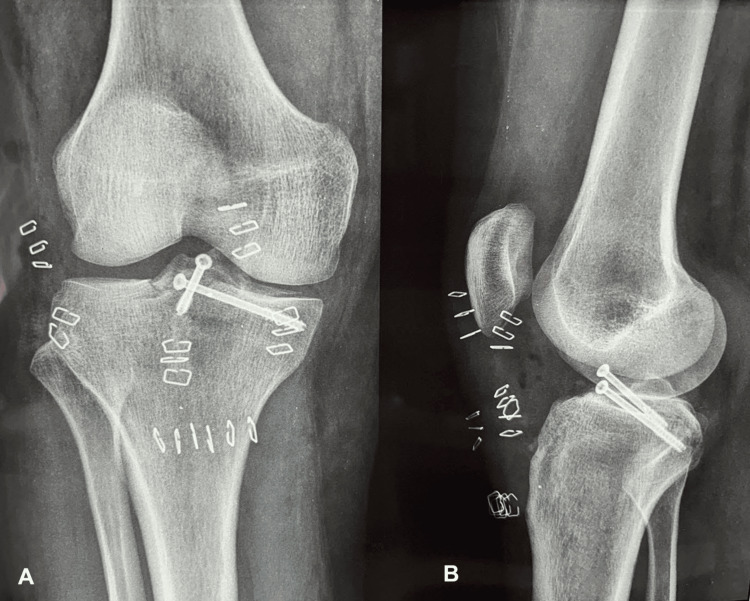
Knee radiographs just after the operation where it can be noticed that the anatomic reduction has been achieved. (A) Anteroposterior view. (B) Lateral view: internal fixation with two antegrade cannulated screws.

A 4 cm longitudinal incision was carried out in the anteromedial tibia, and with the aid of the ACL tibia guide, a 4.5 mm tunnel was conducted by drilling towards the tibial spine and the site of the fracture. All of the suture limbs were brought out from the tibial tunnel with the aid of a probe, and the knee was brought into a 20° of flexion. A biocomposite cannulated interference screw (Milagro Advance Interference Screw 7 × 23 mm DePuy Mitek) was placed through the tibial tunnel for suture fixation in order to provide additional stability. The free limbs of the Orthocord sutures were tied firmly after the interference screw was inserted. Reduction was estimated again by moving the knee to a range of movement from extension to flexion and vice versa, ACL tension was assessed by a probe, and postoperative radiographs were obtained to corroborate retaining of the fracture anatomic reduction (Figure 7).

Rehabilitation

A hinged knee brace locked-in extension was applied to the knee immediately after the operation. The patient was encouraged to perform isometric quadriceps exercises from the first postoperative day, and he initially started to walk with partial weight bearing for the first four weeks. Afterwards, the brace was adjusted to allow the patient's knee flexion up to 90°. Full weight bearing and full range of motion were allowed at six weeks. The patient returned to his daily routine at three months and to his previous sports-related activities after he completed six months of muscle strength, proprioception, and flexibility rehabilitation physiotherapy.

Postoperative clinical evaluation

**Figure 8:**
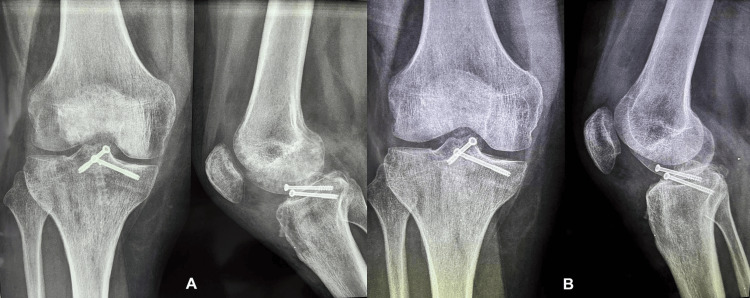
Postoperative (anteroposterior and lateral views) radiographs of the patient's ACL-PCL reconstructed knee. (A) Postoperative X-rays at three months. (B) Postoperative X-rays at six months. Radiological fracture healing can be observed not only at six months but also at three months post-op. ACL: anterior cruciate ligament; PCL: posterior cruciate ligament

The patient was evaluated at 1.5, three, and six months postoperatively (Figure 8A, 8B). Radiographs revealed fracture healing in its anatomical position, and its union was confirmed since no radiographic lines were obtained at the three-month postoperative (post-op) radiograph.

**Figure 9:**
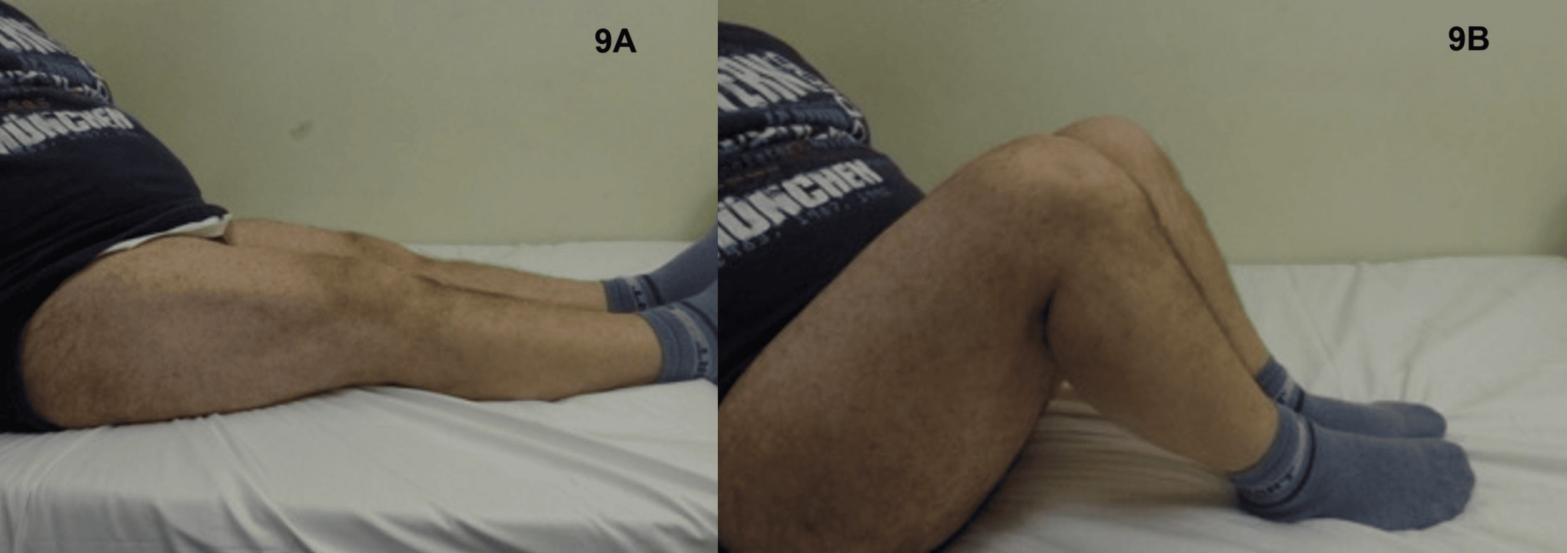
Six months post-op: physical examination. (A) Knee extension. (B) Knee flexion.

Additionally, a comprehensive clinical postoperative examination was performed by using the Lachman, pivot shift, and anterior drawer tests, in order to estimate anteroposterior laxity. Range of motion was assessed with the aid of a goniometer and pain by using the Visual Analogue Scale (VAS) and the International Knee Documentation Committee (IKDC) score, whilst the Hospital for Special Surgery (HSS) and Lysholm score were used to evaluate knee function. All tests for the assessment of anteroposterior laxity were negative. The post-op range of motion displayed a deficit of 5° for flexion and 2° for extension as compared to the uninjured knee (range of motion: 2-125°). VAS score was 0, IKDC score was 90, and HSS and Lysholm scores were 95 and 94, respectively (Figure 9A, 9B).

## Discussion

Tibial eminence fractures are more common in children and adolescents than in adults because of the weakness of their incompletely ossified tibial plateau relative to the strength of their native ACL-PCL [1-6]. However, nowadays, in skeletally mature people, the incidence of these injuries has multiplied because of the increasing population participating in competitive sports activities [1-6]. The mechanism of injury includes hyperextension with simultaneous rotation of the knee on the tibia. This hyperextension places significant tension on the cruciate ligaments, resulting in an avulsion fracture [6]. According to Meyers and McKeever's classification, apart from type I fractures which can be treated conservatively, all the other types with displacement or fragment comminution mostly demand surgical treatment [2,7,10].

The basic aim of surgeons is to avoid non-union or malunion [17]. Screw and suture fixation are the most com­monly described fixation techniques. Open reduction and internal fixation, mostly used in the past, provides anatomic reduction and secure fixation offering direct vision of lesion but causes higher morbidity as compared to arthroscopic techniques [7,13].

Nowadays, the familiarization of orthopaedic community with arthroscopy resulted in the development of arthroscopic techniques for the treatment of tibial eminence fractures. Particularly, arthroscopic osteosynthesis, which might include screw fixation using cannulated cancellous screws [1,2], headless compression screws, or absorbable screws, has offered satisfactory results with decreased postoperative pain, with minimal morbidity and stable fixation for early mobilization despite the higher risk for bone fragmentation and ligament cutting, and without the need of a second operation for hardware removal [2,10-13].

Similar results have been demonstrated by arthroscopic suture fixation systems which might include suture loop transport­ers, multiple drill tunnels, retrograde guides, and various tissue penetrators for suture pas­sage, suture anchors, wires, buttons, or other various systems for meniscal repair with minimal morbidity, no need for hardware removal, and lower risk of bone fragmentation and ligament or neurovascular damage [10-13].

Moreover, in the last decades, arthroscopic surgery as a minimally invasive procedure has also been used for treating avulsion fractures of the PCL with a lower risk of damage to soft tissue and neurovascular bundles at the back of the knee, avoiding posterior repair approaches. In these cases, arthroscopic fixation of bony fragment has been executed successfully with the use of cannulated screws and/or suture fixation systems (wires/threads) [14,18]. The choice of the following method usually depends on the size of the fragment [18].

The current manuscript reports a combined displaced ACL and PCL avulsion fracture in a middle-aged man, which was treated successfully with an all-arthroscopic fixation method. To our knowledge, apart from White et al., there are no other publications in the literature reporting simultaneous displaced avulsion fracture of both ACL and PCL treated arthroscopically, outside of the setting of high-energy trauma [16]. However, in this article, an additional meniscal root avulsion fracture was also found. Furthermore, Nizlan et al. reported a case of a traumatic combined ACL and PCL avulsion fracture as shown in the CT scan [19]. However, arthroscopically, it was noted that the injury was in fact only a PCL avulsion fracture that had been displaced anteriorly, mimicking an ACL avulsion fracture on a CT scan.

Concerning the diagnosis in cases of a cruciate ligament avulsion fracture, knee instability, clinically evaluated, should be checked not only with a full set of knee radiographs including anteroposterior and lateral views but also with advanced imaging methods such as CT scan and MRI in order to confirm the diagnosis, determine the fracture pattern for classification, and set possible treatment options [20].

## Conclusions

The gold standard for fracture healing, restoration of knee stability and pre-injury functional activities, and prevention of osteoarthritis development in patients with a combined ACL and PCL avulsion fracture is the anatomic reduction and stable fixation of fracture. Although the ideal method for the treatment of avulsion fractures has not been defined, arthroscopic surgery can be beneficial even in these complicated fractures. 

## References

[1] Lafrance RM, Giordano B, Goldblatt J, Voloshin I, Maloney M (2010). Pediatric tibial eminence fractures: evaluation and management. J Am Acad Orthop Surg.

[2] Lubowitz JH, Elson WS, Guttmann D (2005). Part II: arthroscopic treatment of tibial plateau fractures: intercondylar eminence avulsion fractures. Arthroscopy.

[3] Kristinsson J, Elsoe R, Jensen HP, Larsen P (2021). Satisfactory outcome following arthroscopic fixation of tibial intercondylar eminence fractures in children and adolescents using bioabsorbable nails. Arch Orthop Trauma Surg.

[4] Liao W, Li Z, Zhang H, Li J, Wang K, Yang Y (2016). Arthroscopic fixation of tibial eminence fractures: a clinical comparative study of nonabsorbable sutures versus absorbable suture anchors. Arthroscopy.

[5] Larsen MW, Garrett WE Jr, Delee JC, Moorman CT 3rd (2006). Surgical management of anterior cruciate ligament injuries in patients with open physes. J Am Acad Orthop Surg.

[6] Woo SL, Hollis JM, Adams DJ, Lyon RM, Takai S (1991). Tensile properties of the human femur-anterior cruciate ligament-tibia complex: the effects of specimen age and orientation. Am J Sports Med.

[7] Griffith JF, Antonio GE, Tong CW, Ming CK (2004). Cruciate ligament avulsion fractures. Arthroscopy.

[8] Meyers MH, McKeever FM (1970). Fracture of the intercondylar eminence of the tibia. J Bone Joint Surg Am.

[9] Zaricznyj B (1977). Avulsion fracture of the tibial eminence: treatment by open reduction and pinning. J Bone Joint Surg Am.

[10] Ahn JH, Yoo JC (2005). Clinical outcome of arthroscopic reduction and suture for displaced acute and chronic tibial spine fractures. Knee Surg Sports Traumatol Arthrosc.

[11] Hirschmann MT, Mayer RR, Kentsch A, Friederich NF (2009). Physeal sparing arthroscopic fixation of displaced tibial eminence fractures: a new surgical technique. Knee Surg Sports Traumatol Arthrosc.

[12] Faivre B, Benea H, Klouche S, Lespagnol F, Bauer T, Hardy P (2014). An original arthroscopic fixation of adult's tibial eminence fractures using the Tightrope® device: a report of 8 cases and review of literature. Knee.

[13] Huang TW, Hsu KY, Cheng CY, Chen LH, Wang CJ, Chan YS, Chen WJ (2008). Arthroscopic suture fixation of tibial eminence avulsion fractures. Arthroscopy.

[14] Guo H, Zhao Y, Gao L (2022). Treatment of avulsion fracture of posterior cruciate ligament tibial insertion by minimally invasive approach in posterior medial knee. Front Surg.

[15] Berrian KM, Lee P, Issack PS (2022). Simultaneous displaced anterior and posterior cruciate ligament avulsion fractures after fall from a bicycle: a case report. JBJS Case Connect.

[16] White CC, Powell CW, Langley C, Bruce JR (2023). A sleepwalking patient presenting with concomitant ACL, PCL, and meniscal root avulsion fractures: a case report. J Orthop Case Rep.

[17] Kendall NS, Hsu SY, Chan KM (1992). Fracture of the tibial spine in adults and children. A review of 31 cases. J Bone Joint Surg Br.

[18] Nakagawa S, Arai Y, Hara K, Inoue H, Hino M, Kubo T (2017). Arthroscopic pullout fixation for a small and comminuted avulsion fracture of the posterior cruciate ligament from the tibia. Knee Surg Relat Res.

[19] Nizlan MN, Suhail A, Samsudin OC, Masbah O (2004). An unusual radiographic presentation of posterior cruciate ligament avulsion fracture. Med J Malaysia.

[20] Gottsegen CJ, Eyer BA, White EA, Learch TJ, Forrester D (2008). Avulsion fractures of the knee: imaging findings and clinical significance. Radiographics.

